# Factors associated with COVID-19 misinformation rebuttal among college students: a descriptive study

**DOI:** 10.3389/fpubh.2023.1233414

**Published:** 2023-11-17

**Authors:** Yi Shan, Meng Ji

**Affiliations:** ^1^School of Foreign Studies, Nantong University, Nantong, China; ^2^School of Languages and Cultures, University of Sydney, Sydney, NSW, Australia

**Keywords:** factors, susceptibility, COVID-19, online misinformation, college students

## Abstract

**Background:**

The deluge of COVID-19 misinformation makes people confused, and acting on such misinformation can kill, leading to the tragic outcome of death. This makes it necessary to identify significant factors associated with college students’ susceptibility.

**Objective:**

This descriptive study sought to ascertain factors significantly associated with college students’ susceptibility to online COVID-19 misinformation.

**Methods:**

To assess college students’ susceptibility to COVID-19 misinformation, we first chose as independent variables some demographic information, some well-developed, validated literacy tools, and the Patient Health Questionnaire-9 Items. Second, we selected as the dependent variable COVID-19 myths from some authoritative, official websites. Third, we integrated the independent and dependent variables into an online questionnaire. Fourth, we recruited students from Nantong University in China to participate in an online questionnaire survey. Finally, based on the data collected, we conducted quantitative and qualitative analyses to relate the independent variables to the dependent variable.

**Results:**

Five hundred forty-six students participated in the survey voluntarily, and all questionnaires they answered were valid. The participants had an average of 2.32 (SD = 0.99) years of higher education. They have a mean age of 20.44 (SD = 1.52) years. 434 (79.5%) of the 546 participants were females. The frequency of their Internet use averaged 3.91 (SD = 0.41), indicating that they logged onto the Internet almost every day. Their self-reported Internet skill was rated 3.79 (SD = 1.07), indicating that the participants rated their Internet skills as basically “good.” The mean scores of the sub-constructs in the AAHLS were 6.14 (SD = 1.37) for functional health literacy, 5.10 (SD = 1.65) for communicative health literacy, and 11.13 (SD = 2.65) for critical health literacy. These mean scores indicated that the participants needed help to read health-related materials “sometimes,” the frequency that they knew how to communicate effectively with professional health providers was between “often” and “sometimes,” and the frequency that they were critical about health information was between “often” and “sometimes,” respectively. The sum of their scores for eHealth literacy averaged 28.29 (SD = 5.31), showing that they had a relatively high eHealth literacy level. The mean score for each question in the GHNT was determined at 1.31 (SD = 0.46), 1.36 (SD = 0.48), 1.41 (SD = 0.49), 1.77 (SD = 0.42), 1.51 (SD = 0.50), and 1.54 (SD = 0.50), respectively. These mean scores showed that a high percentage of the participants answered the 6 questions wrongly, especially Questions 4–6. Similarly, participants performed unsatisfactorily in answering the 3 questions in the CRT, with a mean score of 1.75 (SD = 0.43), 1.55 (SD = 0.50), and 1.59 (SD = 0.49) for each question, respectively. In the PHQ-9, the participants reported that they never felt depressed or felt depressed only for 1–3 days in the past week. The mean score for myths 1–6 and 9–10 ranged from 1.15 (SD = 0.36) to 1.29 (SD = 0.46). This meant that the participants rated these myths false. However, most of the participants rated myths 7–8 true (1.54, SD = 0.50; 1.49, SD = 0.50), showing that they were highly susceptible to these 2 pieces of misinformation. Through data analysis via Logistic Regression (forward stepwise), we found that (1) at an average threshold of 0.5, Internet use frequency, functional health literacy, general health numeracy, reflective thinking tendency, and depression severity were significant predictors of susceptibility to misinformation for both male and female students, (2) at a higher threshold of 0.8, aggregated general health numeracy scores and functional health literacy scores, as well as depression severity were predictors of susceptibility to misinformation for both male and female students, (3) functional health literacy, general health literacy, and depression predicted resistance to misinformation for female students, and (4) internet use frequency and self-reported digital health literacy predicted resistance to misinformation for male students.

**Conclusion:**

We revealed the complexity, dynamics, and differences in age, gender, education, Internet exposure, communicative health literacy, and cognitive skills concerning college students’ susceptibility to online COVID-19 misinformation. Hopefully, this study can provide valuable implications for counteracting COVID-19 misinformation among Chinese college students.

## Introduction

## Background

Misinformation is false information that is shared by people with on intention to mislead others (1). Misinformation often prevails when information gaps or unsettled science motivate people to seek to reason, better understand, and fill in the gaps (1). Misinformation, conspiracy theories, and unverified information on COVID-19 have taken the form of fabricated content and true information that is presented in misleading ways (2–5). The deluge of misinformation makes people confused as to which sources of information are believable (6). Acting on misinformation can kill, leading to the tragic outcome of death (7). According to the statistics of WHO, during the first 3 months of 2020, about 6,000 people across the world were hospitalized and at least 800 died due to COVID-19 misinformation (7).

False information runs the gamut, including discrediting the threat of COVID-19, whether people can use public health measures (e.g., mask-wearing) to protect themselves, erroneous treatments and cures, conspiracy theories that vaccination can change human DNA, etc. (7). Social media platforms significantly contribute to the deluge of misinformation (8). In this context, health organizations across the world have endeavored to curb misinformation. For example, WHO has joined hands with the UK Government to launch an awareness campaign about the risks of misinformation about COVID-19 (9). Currently, WHO is promoting the global campaign “Stop The Spread” to raise people’s awareness about the risks of misinformation on COVID-19, encourage them to double-check information with trusted sources such as WHO and national health authorities, address the infodemic of misinformation on COVID-19, and find and disclose myths about the spread, diagnosis, and treatment of the pandemic (9). WHO promotes infodemic management as the systematic use of risk- and evidence-based analysis and approaches to manage the infodemic and reduce its impact on health behaviors during health emergencies (10). Infodemic management aims to enable good health practices through 4 types of activities: listening to community concerns and questions; promoting understanding of risk and health expert advice; building resilience to misinformation; and engaging and empowering communities to take positive action (10).

In the context of more than 3 billion people using the Internet globally (11), information-seeking is one of the overriding reasons for Internet use, and online information supplements and even replaces data found through traditional sources (12). As reported in a previous study (13), more than 80% of people with a particular health problem have consulted online information about their condition in China. College students tend to be heavy media users (14). Therefore, online health information seeking is of great importance among Chinese college students. However, the digital age has magnified the adverse effects of the current online “infodemic” (15), making it difficult for the public to find trustworthy information among excessive online data (16). There is, therefore, a rampant deluge of incomplete, inaccurate, or false health information in the domain of medicine (17). The mixed quality of online information, easy access to misinformation, and adverse implications of misusing misinformation (12) all make it necessary for us to evaluate the susceptibility to COVID-19 misinformation among Chinese college students.

Although the scientific community has carried out and provided unprecedented access to COVID-19-related studies (18), there is still the prevalence of misinformation on medical topics, which is easily accessible and frequently associated with differential health behaviors, for example, in terms of getting vaccinated, taking herbal supplements, etc. (19). Belief in misinformation on COVID-19 is likely to induce substantive, real-world consequences that make it not only an essential theoretical but also practical field of study (20). Belief in misinformation is not pathological at all, but worthy of being seriously taken as an independent area of scientific research (21). Previous studies have been conducted on factors related to belief in misinformation or conspiracy theories, producing varying and inconsistent findings (20). Belief in misinformation was found to be associated with various sociodemographic features, like low education (22), high education (23), social dynamics (24), age (25), etc. It has also been found to be related to political orientation (25–27). Cognitive sophistication was identified as an effective predictor of the endorsement of misinformation on COVID-19 (28, 29). Some studies also investigated the relationship between religion and endorsement or belief in misinformation (23, 30). Besides, other factors were also found to be contributors to susceptibility to online COVID-19 misinformation, including health-related knowledge, attitudes, and beliefs (17), occupation (31), objective health literacy (32), the efficacy of digital literacy (33), and some information competencies, including information literacy, science literacy, interpersonal trust, and trust in health authority (34). However, these studies did not exclusively investigate college students’ vulnerability to COVID-19 misinformation, they did not examine the role of gender in influencing the study participants’ susceptibility to misinformation, and they did not use some well-developed, validated health literacy tools to capture some of the informants’ demographic characteristics that are supposedly more relevant to their susceptibility to misinformation.

Based on the analysis above and the research gaps identified in particular, we posed some research questions as follows:

*Does college students’ educational level influence their COVID-19 misinformation susceptibility?*

*Is college students’ gender associated with their COVID-19 misinformation susceptibility?*

*What health literacy skills can help college students rebut COVID-19 misinformation?*

## Objective

This descriptive study sought to ascertain factors that were significantly associated with web-based COVID-19 misinformation susceptibility in the cohort of college students. Specifically, we first aimed to integrate into the informants’ demographics some important data captured through some well-developed, validated health literacy tools (specified in the Methods section). The information thus captured was believed to be associated with the informants’ objective health literacy which was found effective in counteracting online misinformation (32). Subsequently, we pinpointed the demographic information most likely contributing to informants’ susceptibility to online COVID-19 misinformation.

## Methods

Although there are several studies recently devoted to the topic, no specific conceptual framework has clearly been stated and used in these studies. Informed by a recent study that investigated susceptibility to breast cancer misinformation among Chinese patients (35), we incorporated into the questionnaire some validated scales, including the All Aspects of Health Literacy Scale (AAHLS) (36), the eHealth Literacy Scale (eHEALS) (37), the General Health Numeracy Test (GHNT-6) (38), the Cognitive Reflection Test (CRT) (39), and the Patient Health Questionnaire-9 Items (PHQ-9) (40). We also followed this study as a conceptual framework, since there was no better alternative, as we stated above. In the absence of an internationally standardized survey tool to assess one’s ability to detect and appraise online misinformation about COVID-19, we adopted a gradient approach to define and quantify the level of misinformation rebuttal among the survey participants. It was achieved by adjusting the threshold of correct responses required for a student to be identified as able to detect general COVID-19 misinformation. Using the aforementioned scales as predictors and adopting Shan et al. (35) as a conceptual framework, this descriptive study sought to pinpoint factors significantly correlated with college students’ susceptibility to Internet-mediated COVID-19 misinformation.

### Questionnaire design

Four parts were included in the questionnaire designed for this study. Part 1 is related to the informants’ age, gender, and education. Part 2 is concerned with the informants’ self-reported Internet skills. Part 3, the highlight of the questionnaire, consists of 5 well-developed, validated health literacy tools (All Aspects of Health Literacy Scale (AAHLS) (36), the eHealth Literacy Scale (eHEALS) (37), the General Health Numeracy Test (GHNT-6) (38), the Cognitive Reflection Test (CRT) (39), and the Patient Health Questionnaire-9 Items (PHQ-9) (40)). Part 4 comprises 10 COVID-19 myths retrieved from some influential, official websites (41–44) of the Centers for Disease Control and Prevention, USA, the Johns Hopkins University School of Medicine, the World Health Organization, and the Australian Department of Health.

Although we submitted the English version of the questionnaire as Supplementary material for better understanding by international readers, the questionnaire was administered in Mandarin Chinese for accurate understanding by the study participants. The English-to-Chinese translation and cultural adaptation of the scales used in the questionnaire was based on a cognitive interview with a small group (10 male and 10 female) of Chinese university students. During the interview, students were invited to review and provide open feedback on the Chinese translation in terms of cultural relevance (whether the scales are relevant to your daily life) and linguistic understandability (whether the scales are comprehensible to you, and whether some terms or expressions are ambiguous to you). Based on the feedback, we improved the translated scales. After that, we repeated the cognitive interview to solicit feedback again. There were three rounds of such reviews and improvements before we finalized the translations.

The highlight, Part 3, was intended to solicit some information on the informants’ objective health literacy, which has the potential to help people tell misinformation (32). The AAHLS (36) is designed to evaluate functional, communicative, and critical health literacy. It identifies the health literacy support needs as well as the strengths and capabilities of an individual and assesses the influence of local patient education initiatives (45, 46). It provides healthcare practitioners with important information on users’ health literacy needs and capabilities (46). The eHEALS (37) measures users’ combined knowledge, comfort, and perceived skills in terms of finding, assessing, and applying electronic health information (47). Reliably and consistently capturing the eHealth literacy concept in repeated administrations, the eHEALS promises to help evaluate user comfort and skills in adopting information technology for health (47). The GHNT-6 (38) is a reliable and valid measure of general health numeracy (48, 49). The CRT (39, 50) has been verified to be an effective scale for assessing individual differences in thinking, judgments, and decisions. It shows substantial correlations with common biases in judgments and decisions (51). The PHQ-9 (40) is an instrument for diagnosing major depressive disorder among adults (52). It has been widely adopted as a screening and diagnostic scale in clinical and population-based research (53, 54) to gauge the severity of depression symptoms. This test was relevant to our investigation based on the clinical experience of 2 researchers (ZX and ZD), who reported an apparent association between patients’ status of depression and their misinformation appraisal skills. Besides, a recent study revealed the association between misinformation exposure and psychological distress including anxiety, depression, and posttraumatic stress disorder symptoms (15). This was another consideration justifying our incorporation of the PHQ-9 into our questionnaire.

### Selection of COVID-19 myths

The myths were selected from the websites of The Centers for Disease Control and Prevention, USA (41), the School of Medicine of Johns Hopkins University (42), the World Health Organization (43), and the Australian Federal Department of Health (44). The selection of these myths was based on the cultural relevance of the statements to everyday life circumstances of university students in China, and the cognitive discernability of myths through a focused group interview with students before we distributed the questionnaire at a larger scale. Myths that were easily detectable by all the students in the interview, such as “COVID-19 vaccines contain magnetic chips,” “vaccines can make me infected,” and “vaccines can change my DNA,” were deleted. We compiled a list of ten myths in Appendix 1 in English from the above-mentioned sources based on our understanding that there were similar or culturally adapted versions of the myths on popular Chinese social media to ensure that the questionnaire was of cultural relevance to the survey participants.

### Recruitment of informants and questionnaire survey

Both undergraduate and graduate students studying at the School of Foreign Studies, Nantong University, were recruited as informants. On the other hand, COVID-19 as a major life stressor impacts their mental well-being directly and indirectly (55). Direct impacts include students’ emotional feelings about COVID-19, such as fear of being infected (56–58). The predefined inclusion criteria comprise (1) being aged 18 years or older and (2) voluntarily participating in the survey. On the one hand, they were heavy media users, like those university students studied by Rideout et al. (14). We made face-to-face contact with students in the form of class meetings to identify those who satisfied the inclusion criteria, explain the purpose of the survey, and ask them to participate in the web-based survey as scheduled. We identified 712 eligible students, who were invited to the project via a web-based link to the questionnaire and the consent form before the survey. They received written information on this study, including the study objective and steps, voluntary participation, and an option of withdrawal during any phase. They were assured of confidentiality and secure data storage.

We conducted a questionnaire survey administered via *wenjuanxing* (59), the most frequently used, influential web-based questionnaire platform in China. The students were asked via email and WeChat groups to answer the online questionnaire anonymously. This online survey lasted 4 days from July 21 to July 24, 2022. Each questionnaire with all questions answered was regarded as valid in this survey.

### Data collection, coding, and analysis

On July 25, 2022, we downloaded the crude data collected through *wenjuanxing* in an Excel form. A total of 546 answered questionnaires were returned, with a response rate of 76.7% (546/712). We double-checked the returned questionnaires and found all of them to be valid. Afterward, we coded the valid data drawing on the predefined coding scheme, to convert text answers into digit answers (scores) for further logistic regression analyses. We then calculated the sums of the scores in the subsections of the AAHLS, and the total scores in the other health literacy tools (eHEALS, GHNT-6, CRT, and PHQ-9) and the 10 COVID-19 myths. Finally, we used Logistic Regression (forward stepwise) in SPSS (v.27) to identify statistically significant factors associated with the ability of Chinese college students to detect and rebut misinformation about COVID-19.

A recent study (35) set the cutoff score for breast cancer misinformation susceptibility at 5 correct answers to the 10 breast cancer-related myths. Informed by this study, we intended to set the cutoff score for COVID-19 misinformation susceptibility at 5 correct answers to the 10 myths about COVID-19. Specifically, if the study participants returned 5 or fewer correct answers to these 10 myths, they were regarded as being susceptible to breast cancer misinformation. Our consultation with health information experts and health educators from Qilu Hospital of Shandong University, China, confirmed the feasibility and rationality of this cutoff score. After identifying factors associated with COVID-19 misinformation susceptibility at this cutoff score, we intended to raise the cutoff score to 8 correct answers to the 10 myths about COVID-19 to further confirm whether we could ascertain the same or similar factors. This raised cutoff score was also deemed rational by the same health information experts and health educators from Qilu Hospital of Shandong University, China. Both authors of this study and all these experts believed that these two cutoff scores could provide a reference for future studies and health education and intervention.

### Assessment of the student participants

We assessed the student participants’ ability to rebut COVID-19 misinformation using logistic regression statistics. Specifically, we chose as independent variables some demographic information of the participants (i.e., age, gender, education, Internet use frequency, and self-reported Internet skills), some validated literacy tools (i.e., AAHLS, eHEALS, and GHNT-6), the Cognitive Reflection Test (CRT), and the Patient Health Questionnaire-9 Items (PHQ-9). We selected as the dependent variable COVID-19 myths from some authoritative, official websites. And then, we used logistic regression statistics to relate the independent variables to the dependent variable. In this way, we identified some essential factors from the independent variables which were statistically significant. These significant factors were used as important indicators of the participants’ COVID-19 misinformation rebuttal ability. In the Results and Discussion sections, we focused on these indicators to assess the students.

### Ethics approval

This study followed the tenets of the Declaration of Helsinki and was approved by the Academic Committee of the School of Foreign Studies, Nantong University, China. Written informed consent was obtained from all study participants who were assured that their responses would remain confidential and anonymous and be only used for academic purposes. We recruited students who were willing to support our research without compensation.

## Results

### Participant descriptive statistics

Participant descriptive statistics are presented in Appendix 2. 546 students participated in the survey voluntarily, and all of their answers were verified to be valid. The participants had an average of 2.32 (SD = 0.99) years of higher education. They have a mean age of 20.44 (SD = 1.52) years. 434 (79.5%) of the 546 participants were females. The frequency of their Internet use averaged 3.91 (SD = 0.41), indicating that they logged onto the Internet almost every day. Their self-reported Internet skill was rated 3.79 (SD = 1.07) according to a 5-point Likert scale (1 = poor, 2 = reasonable, 3 = average, 4 = good, and 5 = excellent), indicating that the participants rated their Internet skill basically “good.” The mean scores of the sub-constructs in the AAHLS were 6.14 (SD = 1.37) for functional health literacy, 5.10 (SD = 1.65) for communicative health literacy, and 11.13 (SD = 2.65) for critical health literacy, in light of a 3-point Likert scale (1 = often, 2 = sometimes, and 3 = rarely). These mean scores indicated that the participants needed help to read health-related materials “sometimes,” the frequency that they knew how to communicate effectively with professional health providers was between “often” and “sometimes,” and the frequency that they were critical about health information was between “often” and “sometimes,” respectively. The sum of their scores for eHealth literacy averaged 28.29 (SD = 5.31) based on a 5-point Likert scale (1 = strongly disagree, 2 = disagree, 3 = undecided, 4 = agree, and 5 = strongly agree), showing that they had a relatively high eHealth literacy level. The mean score for each question in the GHNT was determined at 1.31 (SD = 0.46), 1.36 (SD = 0.48), 1.41 (SD = 0.49), 1.77 (SD = 0.42), 1.51 (SD = 0.50), and 1.54 (SD = 0.50), respectively, based on a 2-point Likert scale with 1 representing a right answer and 2 representing a wrong answer. These mean scores showed that a high percentage of the participants answered the 6 questions wrongly, especially Questions 4–6. Similarly, participants performed unsatisfactorily in answering the 3 questions in the CRT, with a mean score of 1.75 (SD = 0.43), 1.55 (SD = 0.50), and 1.59 (SD = 0.49) for each question respectively, based on a 2-point Likert scale with 1 representing a right answer and 2 representing a wrong answer. In the PHQ-9, the participants reported that they never felt depressed or felt depressed only for 1–3 days in the past week.

### Statistics of student responses to the 10 myths about COVID-19

Multimedia Appendix 2 presents the statistics of student responses to the 10 myths about COVID-19. The mean score for myths 1–6 and 9–10 ranged from 1.15 (SD = 0.36) to 1.29 (SD = 0.46). This meant that the participants rated these myths false. However, most of the participants rated myths 7–8 true (1.54, SD = 0.50; 1.49, SD = 0.50), showing that they were highly susceptible to these 2 pieces of misinformation.

### Multilinearity statistics of the 12 predictor variables in the regression model

Table 1 shows the multilinearity statistics of the 12 predictor variables in the regression model. It shows that all variables had a Variance Inflation Factor (VIF) under or slightly above 2, widely used as the threshold for acceptable multilinearity for regression in the literature (60, 61). Small VIFs are indicative of limited, tolerable correlation among the pre-selected predictor variables (12 in total in our study) which are required to develop logistic regression models of higher generalisability and reliability.

**Table 1:** Multilinearity statistics.

Predictor variables ^a^	Tolerance	VIF
(Constant)		
Years of HE	0.71	1.4
Age	0.7	1.42
Gender	0.92	1.09
Internet use frequency	0.96	1.04
Self-assessed internet skill	0.93	1.07
FHL-SUM	0.7	1.43
COHL-SUM	0.48	2.09
CRHL-SUM	0.52	1.94
eHEALS-SUM	0.9	1.11
GHNT-SUM	0.58	1.72
CRT-SUM	0.61	1.65
PHQ9-SUM	0.97	1.04

^a^ Years of HE, Years of Higher Education; FHL-SUM, functional health literacy sum scores; COHL-SUM, communicative health literacy sum scores; CRHL-SUM, critical health literacy sum scores; eHEALS-SUM, digital health literacy sum scores; GHNT-SUM, General Health Literacy Test sum scores; CRT-SUM, Cognitive Recognition Test sum scores; PHQ9-SUM, PHQ-9 Patient Depression Questionnaire sum scores.

### Thresholds of COVID-19 misinformation rebuttal

In this subsection, we present the result of the logistic regression analyses (forward stepwise) of the internal and external factors associated with one’s ability to rebut COVID-19 myths when the threshold increased from an average level of 0.5 (having 5 or more correct responses) to 0.8 (having 8 or more correct responses) to understand the complexity, variability, as well as gendered differences in discerning and invalidating online misinformation about the pandemic among Chinese university students.

Table 2 shows the factors associated with the ability to detect and rebut online COVID-19 misinformation when the threshold of correct responses to the 10 myths was set at 0.5. It means that a student needs to identify 5 or more myths to reach the qualifying threshold. Table 2 shows the regression model developed on the entire dataset including both genders. It shows that the number of years of university education (Years of HE) was a statistically significant factor (OR = 1.54, CI [1.12, 2.11], *p* = 0.01). With 1 year more university education, the odds of a student being able to reach the misinformation rebuttal threshold increased by 54%. Internet usage frequency was another significant indicator. The original questionnaire contained 4 ordinal levels for Internet Use Frequency (1 = rarely, 2 = once a week, 3 = a few days a week, and 4 = almost every day of the week). Table 2 shows that Internet Use (2) and (3) predicted statistically significant decreases in the odds of students being able to discern and rebut COVID-19 myths: Internet Use (2 = once a week) (OR = 0.14, CI [0.02, 1.07], *p* = 0.06), Internet Use (3 = a few days a week) (OR = 0.25, CI [0.09, 0.69], *p* = 0.01). This means that when the frequency of internet usage changed from ‘almost every day’ to ‘a few days a week’, or to ‘once a week’ the odds of a student being capable to detect COVID-19 myths on the social media dropped by 75 and 86%, respectively. It was revealing to notice that despite a large decrease of 56% in the odds of being capable of detecting online COVID-19 myths when the internet use profile of a student changed from ‘almost every day’ to ‘rarely,’ such difference was not statistically significant (*p* = 0.37). This seems to suggest that it was the infrequent, limited access to the Internet which constituted significant risks to online health myths differentiation among Chinese college students, rather than high-level exposure (‘almost every day’) or minimal exposure (‘rarely’) to the internet.

**Table 2:** Factors associated with the ability to detect COVID-19 misinformation (Threshold = 0.5, Gender = Both).

Predictors	*B*	S.E.	Wald	df	Sig.	Exp(B)	95% C.I. for EXP(B)	
							Lower	Upper
Years of HE	0.43	0.16	7.14	1.00	0.01	1.54	1.12	2.11
Internet use			10.91	3.00	0.01			
Internet use (1)	−0.89	0.99	0.82	1.00	0.37	0.41	0.06	2.83
Internet use (2)	−1.94	1.02	3.60	1.00	0.06	0.14	0.02	1.07
Internet use (3)	−1.40	0.52	7.21	1.00	0.01	0.25	0.09	0.69
FHL1			6.89	2.00	0.03			
FHL1(1)	−1.11	0.43	6.68	1.00	0.01	0.33	0.14	0.77
FHL1(2)	−0.31	0.34	0.81	1.00	0.37	0.74	0.38	1.44
eHEALS-SUM	0.05	0.02	4.73	1.00	0.03	1.05	1.01	1.09
GHNT-SUM	−0.27	0.11	6.12	1.00	0.01	0.76	0.62	0.95
CRT-SUM	−0.70	0.23	9.04	1.00	0.00	0.50	0.32	0.79
PHQSUM	−0.05	0.02	3.98	1.00	0.05	0.95	0.91	1.00
Constant	6.77	1.44	22.19	1.00	0.00	873.84		

Functional health literacy refers to one’s ability to seek and understand health information (62). It contains three questions: FHL1: How often do you need someone to help you when you are given information to read by your doctor, nurse, or pharmacist? FHL2 When you need help, can you easily get hold of someone to assist you? FHL3: Do you need help to fill in official documents? (46). Each question has three frequency levels which we coded as ordinal data in our study: 1 = often, 2 = sometimes, 3 = rarely. As a result, larger scores on each question imply more limited functional literacy required to identify and comprehend health information. Regression modeling in Table 2 shows that when the frequency of a student needing others’ help to read and understand a piece of health information increased, the odds of the student being able to detect COVID-19 myths dropped significantly, especially when the frequency of seeking help to comprehend health information increased from ‘rarely’ to ‘often’: FHL1(1) (OR = 0.33, CI [0.14, 0.77], *p* = 0.01). However, when frequency increased from ‘rarely’ to ‘sometimes,’ the odds of the student being able to detect COVID-19 myths did not differ significantly from that of students rarely needing others’ help to understand health information: FHL1(2) (OR = 0.74, CI [0.38, 1.44], *p* = 0.37).

Digital health literacy (eHEALS) proved another significant predictor of students’ ability to detect and rebut COVID-19 myths. It contains 8 highly related questions that enable a reflective self-assessment of one’s ability to seek, appraise, and utilize quality online health information (47). Each question of the eHEAL scale has five frequency levels which were coded in our study as 1 = highly disagree, 2 = disagree, 3 = not sure, 4 = agree, and 5 = highly agree. To generate the combined scores, we produced the sum of the 8 questions and coded the sum as eHEALS-SUM. Higher combined scores thus indicated greater digital health literacy, as the respondent showed higher levels of confidence in internet usage. Table 2 shows that eHEALS-SUM (OR = 1.05, CI [1.01, 1.09], *p* = 0.03) positively predicted the odds of a student being able to detect COVID-19 myths. With one unit increase in the eHEALS-SUM score, the odds of the student being able to detect COVID-19 myths from the list provided increased by 5%.

In our study, we used two scales to measure cognitive ability to process health and general information which were the General Health Numeracy Test (GHNT), and the Cognitive Recognition Test (CRT). The GHNT-6 was developed to estimate the overall health numeracy skills of patients to understand and act on numerical health information (48, 63). The short form of the test contains 6 questions related to simple calculations of health risks (GHNT1 and GHNT2 on seasonal influenza, GHNT3 heart rate during physical exercise, GHNT4 nutrition composition, GHNT5 relation between cholesterol medication and heart attack risks, and GHNT6 interpretation of positive breast cancer screening test results). In our study, we coded the responses from students as binary data: 1 = correct, and 2 = wrong. As a result, higher sum scores of GHNT are indicative of more reduced general health numeracy. Logistic regression modeling in Table 2 shows that the sum score of GHNT was a significant predictor of student’s ability to detect and rebut myths about COVID-19: GHNT-SUM (OR = 0.76, CI [0.62, 0.95], *p* = 0.01). This means with the increase of one unit in the aggregated score of GHNT (having made one more mistake in the overall answers), the odds of the student being able to detect 5 or more misleading statements about COVID-19 in the list dropped by about a third, i.e., 34%. In other words, with one unit decrease in the overall GHNT score (having made one less mistake in the GHNT test), the odds of the student being able to successfully rebut 5 or more myths that we provided to him or her increased by 31.58%.

The Cognitive Recognition Test (CRT) was developed to estimate the cognitive tendency to engage in reflective, contemplative thinking, as opposed more intuitive, hasty thinking style of individuals to reach instinctive ‘gut’ responses. The test contains 3 short questions on the cost of sporting goods, production speed of widgets, and growth rate of lily pads. We coded the responses from students as binary data: 1 = correct and 2 = wrong. The result of the CRT was similar to that of the GHNT. Higher aggregated CRT scores suggest a greater tendency toward more intuitive or less reflective cognitive processing of numerical information. CRT-SUM proved a significant predictor of the student’s capability to detect and rebut myths about COVID-19: CRT-SUM (OR = 0.5, CI [0.32, 0.79], *p* < 0.001). With the increase of one unit (having made one more mistake in the CRT test), the odds of the student being able to detect 5 or more COVID-19 myths decreased by 50%.

Tables 3, 4 show the gender differences in detecting and rebutting COVID-19 myths among the students. For Chinese female university students, significant predictors of capability to detect COVID-19 myths were Years of Higher Education (OR = 1.87, CI [1.25, 2.8], *p* < 0.001), FHL1- the first question of the functional health literacy scale of AAHLS (need to ask help to comprehend health information) (OR = 0.29, CI [0.10, 0.79], *p* = 0.02), the aggregated scores of the GHNT test (OR = 0.75, CI [0.58, 0.97], *p* = 0.03) and CRTSUM (OR = 0.36, CI [0.20, 0.65], *p* < 0.001). Specifically, regarding FHL1, when the frequency of seeking others’ help to comprehend health information increased from ‘rarely’ to ‘often’, the odds of the student reaching the predefined threshold (0.5) of being capable of differentiating COVID-19 myths decreased by 71%. Lower cognitive skills as indicated by higher scores on the GHNT and the CRT scales also predicted significant decreases in the odds of students being able to detect popular COVID-19 myths. For example, with one unit increase (having made one more mistake) on the GHNT and the CRT tests, the odds of the student not being able to rebut 5 or more common COVID-19 myths from the list increased by 33 and 178%, respectively.

**Table 3:** Factors associated with the ability to detect COVID-19 misinformation (Threshold = 0.5, Female).

Predictors	*B*	S.E.	Wald	df	Sig.	Exp(B)	95% C.I. for EXP(B)	
							Lower	Upper
Years of HE	0.63	0.21	9.20	1.00	0.00	1.87	1.25	2.80
FHL1			5.81	2.00	0.06			
FHL1(1)	−1.25	0.52	5.77	1.00	0.02	0.29	0.10	0.79
FHL1(2)	−0.44	0.41	1.13	1.00	0.29	0.64	0.29	1.45
GHNTSUM	−0.29	0.13	4.79	1.00	0.03	0.75	0.58	0.97
CRTSUM	−1.02	0.30	11.48	1.00	0.00	0.36	0.20	0.65
PHQSUM	−0.06	0.03	4.33	1.00	0.04	0.94	0.88	1.00
Constant	9.79	1.81	29.38	1.00	0.00	17929.66		

**Table 4:** Factors associated with the ability to detect COVID-19 misinformation (Threshold = 0.5, Male).

	*B*	S.E.	Wald	df	Sig.	Exp(B)	95% C.I. for EXP(B)	
							Lower	Upper
Internet use (1)	−1.10	1.32	0.70	1.00	0.40	0.33	0.03	4.38
Internet use (2)	−3.16	1.03	9.39	1.00	0.00	0.04	0.01	0.32
Constant	1.55	0.50	9.52	1.00	0.00	4.73		

Another useful finding was that depression was another significant predictor of female students’ performance on COVID-19 myth rebuttal. We estimated the mental health status of students using the PHQ-9 (Patient Depression Questionnaire-9). The scale has 9 correlated questions on self-reported depression severity (53). Each question has four levels of occurrence of depressive symptoms, which we coded as 0 = not at all, 1 = several days, 2 = more than half the days, and 3 = nearly every day. The result shows that with one unit increase in the aggregated score of PHQ9, which indicates a higher level of depression, the odds of the student being capable of successfully detecting 5 or more COVID-19 myths were reduced by 6%.

Factors influencing the performance of male Chinese university students in detecting COVID-19 myths were distinct from those of their female peers. Table 4 shows that limited internet use predicted substantial decreases in the odds of Chinese male college students being able to detect COVID-19 myths. But statistically significant drops in the odds of pandemic myth rebuttal only occurred when the frequency of internet usage changed from ‘almost every day’ to ‘a few days a week’ Internet Use (2) (OR = 0.04, CI [0.01, 0.32], *p* < 0.001). This means that when a male student had access to the internet a few days a week rather than every day of the week, the odds of that male student being able to reach the myth discrimination threshold decreased by as large as 96%. By contrast, we were surprised to find out that the difference between male students who had daily access to the internet and those who used internet only once a week was not statistically significant: Internet Use (1) (OR = 0.33, CI [0.03, 4.38], *p* = 0.4). This finding prompted us to speculate that among Chinese male college students, it was limited access, rather than daily or sporadic access to the internet which constituted a leading risk of students’ vulnerability to online pandemic myths.

When raising the threshold from the average of 0.5 to a higher level of 0.8, we identified a similar but reduced set of factors that were significant predictors of students’ performance in detecting online pandemic myths. Table 5 shows that first, increases in the aggregated score of the functional health literacy scale (FHL-SUM) predicted greater odds of students being able to reach the higher threshold of 0.8, namely, having the capability to correctly detect, rebut 8 or more items about COVID-19 from the list of myths we provided to them: FHL-SUM (OR = 1.21, CI [1.07, 1.38], *p* < 0.001). Recalling that we coded the three-level frequency of the three component questions of FHL in this order: 1 = often, 2 = sometimes, 3 = rarely, a higher aggregated FHL score indicates that an individual is less dependent on others’ help to understand health materials properly (FHL1), more efficient in soliciting support when in need (FHL2), and less reliant on others’ help to complete official medical documents (FHL3). Table 5 shows that with one unit increase in the sum of FHL scores, the odds of a student being able to correctly identify 8 or more myths increased by 21%.

**Table 5:** Factors associated with the ability to detect COVID-19 misinformation (Threshold = 0.8, Both Genders).

	*B*	S.E.	Wald	df	Sig.	Exp(B)	95% C.I. for EXP(B)	
							Lower	Upper
FHLSUM	0.19	0.07	8.53	1.00	0.00	1.21	1.07	1.38
GHN3(1)	0.41	0.20	4.06	1.00	0.04	1.51	1.01	2.25
GHN5(1)	0.49	0.20	5.93	1.00	0.02	1.64	1.10	2.43
PHQSUM	−0.05	0.02	10.03	1.00	0.00	0.95	0.92	0.98
Constant	−1.06	0.44	5.69	1.00	0.02	0.35		

Greater general health numeracy as measured by the GHNT test predicts better performance in myths rebuttal. Two items on the GHNT scale emerged as significant predictors: GHNT3 (1 = correct response) (OR = 1.51, CI [1.01, 2.25], *p* = 0.04) and GHNT5 (1 = correct response) (OR = 1.64, CI [1.10, 2.43], *p* = 0.02). GHNT3 was formulated in the context of calculating the maximal heart rate of older adult females when doing physical exercises, and GHNT5 was related to estimating changes in the probability of heart attacks after taking cholesterol medications for 5 years. Results in Table 5 show that when a Chinese college student correctly answered GHNT3 and GHNT5, the odds of the student being capable of detecting 8 or more pandemic myths from the list increased by 51 and 64%, respectively.

Depression again proved a significant predictor of lower performance in rebutting pandemic myths: PHQ-SUM (OR = 0.95, CI [0.92, 0.98], *p* < 0.001). With one score increase in the aggregated PHQ scores, which is indicative of more severe depression, the odds of students being capable of detecting 8 or more myths decreased by 5%. In our study of Chinese college students, the significant negative impact of depression was confirmed in both scenarios of average and higher-level thresholds of online health myth rebuttal capability.

Tables 6, 7 show gendered differences in detecting pandemic myths among Chinese college students. Table 6 shows that among female students, greater functional health literacy (FHL-SUM) (OR = 1.22, CI [1.04, 1.44], *p* = 0.02), greater general health numeracy (GHNT-SUM) (OR = 0.83, CI [0.74, 0.92], *p* < 0.001) were significant predictors of increased odds of better performance of myth rebuttal, whereas more severe depression (PHQ-SUM) (OR = 0.94, CI [0.91, 0.98], *p* < 0.001) predicted worse outcomes in myths detection among Chinese female students. Specifically, with a unit increase in the aggregated FHL scores, the odds of female students being able to detect 8 or more pandemic myths out of the 10 myths provided increased by 22%. With the increase of one more mistake in the GHNT test, the odds of female students being able to reach the higher myth rebuttal threshold were reduced by 17%. Lastly, with a unit increase on the PHQ depression severity scale, the odds of female students falling under the threshold increased by 6.38%.

**Table 6:** Factors associated with the ability to detect COVID-19 misinformation (Threshold = 0.8, Female).

	*B*	S.E.	Wald	df	Sig.	Exp(B)	95% C.I. for EXP(B)	
							Lower	Upper
FHLSUM	0.20	0.08	5.92	1.00	0.02	1.22	1.04	1.44
GHNTSUM	−0.19	0.06	11.75	1.00	0.00	0.83	0.74	0.92
PHQSUM	−0.06	0.02	9.69	1.00	0.00	0.94	0.91	0.98
Constant	1.12	0.73	2.38	1.00	0.12	3.06		

**Table 7:** Factors associated with the ability to detect COVID-19 misinformation (Threshold = 0.8, Male).

	*B*	S.E.	Wald	df	Sig.	Exp(B)	95% C.I. for EXP(B)	
							Lower	Upper
eHEALS-SUM	0.06	0.03	5.11	1.00	0.02	1.06	1.01	1.12
Constant	−1.44	0.79	3.32	1.00	0.07	0.24		

By contrast, among Chinese male students, it was their self-reported digital health literacy measured by the eHEALS scale that predicted the odds of male students being capable of reaching the higher pandemic myth rebuttal threshold: eHEALS-SUM (OR = 1.06, CI [1.01, 1.12], *p* = 0.02). The digital health literacy scale contains 8 correlated questions enabling a reflective self-assessment of seek, appraise, and utilize critically and effectively online health resources (46). Each question is associated with a 5-item Likert scale which we coded as 1 = strongly disagree, 2 = disagree, 3 = not sure, 4 = agree, and 5 = strongly agree. Higher aggregated eHEALS scores are indicative of greater confidence in internet use for health purposes. Table 7 shows that among Chinese male students, with one unit increase in their aggregated eHEALS scores, the odds of students being capable of successfully detecting 8 or more myths increased by 6%.

## Discussion

### Principal findings in comparison with related publications

*Finding 1: Education, Internet Use Frequency, Functional Health Literacy, General Health Numeracy, Reflective Thinking Tendency, and Depression Severity Were Predictors of Susceptibility to Misinformation about COVID-19 for Both Male and Female Students.*

At an average threshold of 0.5, for both genders, years of higher education, internet use frequency (one day a week and a few days a week), functional health literacy, particularly the first item of the FHL scale (often needing others’ help to understand health materials), general health numeracy, reflective thinking tendency, and depression severity were significant predictors of the capability of detecting popular myths about the pandemic among Chinese college students.

We found that the number of years of university education was a statistically significant factor (OR = 1.54, CI [1.12, 2.11], *p* = 0.01). With 1 year more university education, the odds of a student being able to reach the misinformation rebuttal threshold increased by 54%. This is in tune with previous studies which identified the association between low and high education levels and belief in misinformation (22, 23). However, a recent study dismissed education levels as a predictor of susceptibility to misinformation (20). Therefore, the role of education level in predicting misinformation susceptibility needs to be further ascertained.

COVID-19 is accompanied by an “infodemic,” an overabundance of valid and invalid COVID-19-related information (64, 65). Digital communication technologies, the Internet and social media in particular, allow the COVID-19 infodemic to spread faster than the coronavirus itself (66). As a result, frequent exposure to the Internet increases the possibility of Chinese college students’ vulnerability to COVID-19 misinformation.

We found health literacy, including functional health literacy and general health numeracy, an important predictor of Chinese college students’ rebuttal to COVID-19 misinformation. This finding confirms the findings reported by some previous studies. Health literacy, the ability to seek, comprehend, assess, and apply health information in daily health behaviors and decisions (67), is crucially significant during COVID-19 (68). It has become a core capacity that people need to have for navigating online information and health service environments in the context of COVID-19 and the associated infodemic (69). People with poor health literacy are most probably confused when facing massive amounts of information on the Internet or media (70).

Reflective thinking was found to be an effective factor in predicting ‘students’ capability to detect and rebut COVID-19 misinformation in our study. COVID-19 increasingly demands people to find relevant information, critically reflect on it, and apply it to daily life and practices (66). Cognitive reflection (39, 50) results in individual differences in reflective thinking, judgments, and resistance to making ‘gut’ decisions. It shows substantial correlations with common biases in judgments and decisions (51). Cognitive sophistication (e.g., analytic thinking, basic science knowledge) has been found to effectively predict the endorsement of misinformation on COVID-19 (28), with lower analytic thinking abilities closely associated with the failure to distinguish between true and false news (29).

To our knowledge, no previous studies have investigated the relationship between depression severity and susceptibility to misinformation. Although we identified depression severity as a predictor of Chinese college students’ susceptibility to COVID-19 misinformation, we cannot compare this finding with the findings reported by related publications.

*Finding 2: Aggregated General Health Numeracy Scores and Functional Health Literacy Scores, as well as Depression Severity, Were Predictors of Susceptibility to Misinformation for Both Male and Female Students.*

At a higher threshold of 0.8, for both genders, aggregated functional health literacy scores and general health numeracy scores, as well as depression severity were significant predictors of capability to detect popular myths about the pandemic among Chinese college students.

As an essential component of literacy, numeracy reflects the ability to understand and use quantitative health information in everyday life (63). It is less likely for people with limited health literacy or numeracy to utilize health services effectively (71, 72). It is more likely for people with low health numeracy to experience difficulties in acting on medical instructions (73) comprehending health information (74), and engaging in self-care activities (75, 76), and to experience worse health outcomes (46, 77).

Functional health literacy, including individuals’ abilities to seek and comprehend health-related knowledge (17, 32, 34), was also found to be an effective predictor of students’ capability to rebut online misinformation about the pandemic.

*Finding 3: Functional Health Literacy, General Health Literacy, and Depression Predicted Resistance to Misinformation for Female Students.*

For Chinese female college students, functional health literacy, general health literacy, as well as depression were significant predictors of female students’ capability to detect popular myths about the pandemic.

*Finding 4: Internet Use Frequency and Self-reported Digital Health Literacy Predicted Resistance to Misinformation for Male Students.*

For Chinese male college students, it was their internet use frequency and self-reported digital health literacy that were significant predictors of male students’ capability to detect popular myths about the pandemic. Digital health literacy applies health literacy (67) to digital contexts and environments (78). It is a vital necessity for heavy media users (14) to rebut online misinformation (33). People reported difficulties in dealing with health-related information due to limited digital health literacy (70, 79).

### Implications

This descriptive study can add to the body of evidence supporting the necessity of investigating COVID-19 misinformation rebuttal. Important implications can be provided for clinical practice, health education, medical research, and public health policy-making. The 4 principal findings concerning the predictors of susceptibility to COVID-19 misinformation identified in the study could be used as important indicators for screening those susceptible to COVID-19 misinformation to deliver targeted education and interventions. Knowledge and skills related to the 4 predictors should be integrated into public health education about COVID-19 misinformation to improve the general public’s ability to appraise and rebut COVID-19-related myths. Medical researchers may gain insights into the topic of the susceptibility to COVID-19 misinformation. As a result, they could verify the contributors to COVID-19 misinformation susceptibility ascertained in this study and identify more contributing factors in future studies. Public health policymakers can consider the results and findings of this study when making public health policies in the future.

In the digital age, the mixed quality of online information, easy access to misinformation, and adverse implications of using misinformation all make it essential to evaluate susceptibility to misinformation in the public. Such evaluations can contribute to more tailor-made and targeted infodemic management. As Dr. Tedros Adhanom Ghebreyesus, WHO Director-General, said, “Finding solutions to the infodemic is as vital for saving lives from COVID-19 as public health measures, like mask-wearing and hand hygiene, to equitable access to vaccines, treatments and diagnostics” (7). Given that effective and timely evaluation of COVID-19 misinformation susceptibility can be made in various populations, infodemic management is most likely to enable good health practices through such measures as listening to community concerns and questions, promoting understanding of risk and health expert advice, building resilience to misinformation, and engaging and empowering communities to take positive action (10).

### Limitations

This study has some limitations. First and foremost, it is unclear to what extent a single university sample is representative in the Chinese context. Such representativeness needs to be further attested. The generalizability of the research findings based on such a sample also needs to be further tested. In future studies, we will involve more students from diverse universities across China to test the representativeness of the sample we used in this study and the generalizability of the research findings reported in this study. In this way, we can ascertain more diversified and more tailor-made factors specific to the Chinese college students sample. Second, female participants (79.5%) were far more than male participants in this cross-sectional survey. This may induce a certain level of gender bias, which most probably caused higher self-reported Internet access frequencies and more wrong answers to the question items on the GHNT and CRT scales. These gender bias-induced results may undermine the generalizability of the research findings to some extent. However, our sample reflected a population that is theoretically relevant to key literature on medical misinformation. In future studies, we will try to balance the proportions of male and female participants to minimize gender bias. Third, the assessment of students’ capability of pandemic myth rebuttal was subject to the deliberate selection of thresholds that would suit the varying practical needs of the tool. When setting the threshold at different values, we obtained different findings, as evidenced by principal findings 1 and 2 above. Fourth, the absence of statistically significant differences in differentiating online pandemic myths between male students who had infrequent, limited access to the Internet and those who had high-level exposure (‘almost every day’) to the Internet warrants further research. In comparison, greater exposure to the Internet increased female students’ susceptibility to misinformation about the pandemic. Whether this gender difference may apply to college students’ vulnerability to other online misinformation needs to be ascertained in future studies. Finally, the cross-sectional nature of this study may cause some biases, including a non-response bias, a reporting bias, etc. According to established practice, a non-response rate of over 30% can cause a non-response bias in a questionnaire survey. The response rate (76.7%, 546/712) of the study participants indicates that our cross-sectional study was less likely to be influenced by a non-response bias. However, in the PHQ-9, the participants reported that they never felt depressed or felt depressed only for 1–3 days in the past week. This self-reported depression level was likely to be influenced by a reporting bias because students, especially females, usually prefer not to acknowledge their depressive mood to others. In future studies, we would recruit more cohorts from more walks of life as participants to reduce cross-sectional study-induced biases.

## Conclusion

In this descriptive study, we revealed the complexity and dynamics concerning Chinese college students’ susceptibility to COVID-19 misinformation. Specifically, we found that (1) at an average threshold of 0.5, Internet use frequency, functional health literacy, general health numeracy, reflective thinking tendency, and depression severity were predictors of susceptibility to misinformation for both male and female students, (2) at a higher threshold of 0.8, aggregated general health numeracy scores and functional health literacy scores, as well as depression severity were predictors of susceptibility to misinformation for both male and female students, (3) functional health literacy, general health literacy, and depression predicted resistance to misinformation for female students, and (4) internet use frequency and self-reported digital health literacy predicted resistance to misinformation for male students. It was the first study that assessed Chinese college students’ susceptibility to COVID-19 misinformation through a comprehensive survey of their various health and digital health literacy and skills. This study provided valuable insights into the mechanism of how Chinese students engage or disengage with COVID-19 misinformation We will perform similar studies to assess susceptibility to other health misinformation and disinformation among Chinese college students to identify more contributors to their vulnerability to online misinformation.

## Data availability statement

The raw data supporting the conclusions of this article will be made available by the authors, without undue reservation.

## Ethics statement

The studies involving humans were approved by the Academic Committee of the School of Foreign Studies, Nantong University, China. The studies were conducted in accordance with the local legislation and institutional requirements. Written informed consent for participation in this study was provided by the participants’ legal guardians/next of kin.

## Author contributions

MJ and YS made the data analysis, and designed and wrote this article. YS conducted the survey and collected the data. All authors read and approved the final manuscript.

## Glossary

AAHLS: the All Aspects of Health Literacy Scale
eHEALS: the eHealth Literacy Scale
GHNT-6: the General Health Numeracy Test
CRT: the Cognitive Reflection Test
PHQ-9: the Psychological Health Questionnaire-9 Items
HE: higher education
FHL: functional health literacy
CRHL: critical health literacy
COHL: communicative health literacy
FHL-SUM: sum of the functional health literacy scale of the All Aspects of Health Literacy Scale (AAHLS)
CRHL-SUM: sum of the critical health literacy scale of the AAHLS
COHL-SUM: sum of the communicative health literacy scale of the AAHLS
eHL-SUM: sum of the digital health literacy scale
GHNT-SUM: sum of the general health numeracy scale
CRT-SUM: sum of the cognitive recognition test
PHQ-SUM: sum of the Patient Health Questionnaire-9 (PHQ9)
