# Supplementary material for: Factors associated with COVID-19 misinformation rebuttal among college students: a descriptive study
Source: Front Public Health. 2023 Nov 17;11:1233414. doi: 10.3389/fpubh.2023.1233414 (PMC10690778; doi:10.3389/fpubh.2023.1233414)
Supplement: Supplementary file 1 [file Data_Sheet_1.docx]

**问卷**

**I Basic Information**

**1、Age**

I’m ——years old.

1. **Gender**

- male □
- female □

**3、Education**

- freshman □
- sophomore □
- junior □
- senior □
- first-year graduate □
- second-year graduate □
- third-year graduate □

**II Information about Logging onto the Internet**

**1、Frequency of logging onto the Internet**

- Almost everyday □
- Several days a week □
- One day a week □
- Basically never □

**2、Ways of logging onto the Internet**

- mobile □
- Portable PC □
- Home computer □
- Ipad □
- Office computer □
- Public computer □

**3、Self-reported skills in logging onto the Internet**

- Very good □
- Good □
- OK □
- Not bad □
- poor □

**III Health Literacy**

**1、All Aspects of Health Literacy**

Please tick one response only for each question by placing a tick in the box. If you prefer, a member of staff or the research team can read out questions to yo.

1）How often do you need someone to help you when you are given information to read by your doctor, nurse or pharmacist?

□ often □ sometimes □ rarely

2）When you need help, can you easily get hold of someone to assist you?

□ often □ sometimes □ rarely □ not applicable

3）Do you need help to fill in official documents?

□ often □ sometimes □ rarely

4）When you talk to a doctor or nurse, do you give them all the information they need to help you?

□ often □ sometimes □ rarely

5）When you talk to a doctor or nurse, do you ask the questions you need to ask?

□ often □ sometimes □ rarely

6）When you talk to a doctor or nurse, do you make sure they explain anything that you do not understand?

□ often □ sometimes □ rarely

7）Are you someone who likes to find out lots of different information about your health?

□ often □ sometimes □ rarely

8）How often do you think carefully about whether health information makes sense in your particular situation?

□ often □ sometimes □ rarely

9）How often do you try to work out whether information about your health can be trusted?

□ often □ sometimes □ rarely

10）Are you the sort of person who might question your doctor or nurse’s advice based on your own research?

□ yes, definitely □ maybe/ sometimes □ not really

11）Do you think that there plenty of ways to have a say in what the government does about health?

□ yes, definitely □ maybe/ sometimes □ not really

12）What do you think matters most for everyone’s health? (tick one answer only)

□ a) information and encouragement to lead healthy lifestyles

□ b) good housing, education, decent jobs and good local facilities

**2、eHealth Literacy**

Please tick one response only for each question by placing a tick in the box.

1. I know what health resources are available on the internet.

□ strongly disagree □ disagree □ neither agree nor disagree　　□ agree □ strongly agree

1. I know where to find helpful health resources on the internet.

□ strongly disagree □ disagree □ neither agree nor disagree　　□ agree □ strongly agree

1. I know how to find helpful health resources on the internet.

□ strongly disagree □ disagree □ neither agree nor disagree　　□ agree □ strongly agree

1. I know how to use the internet to answer my health questions.

□ strongly disagree □ disagree □ neither agree nor disagree　　□ agree □ strongly agree

1. I know how to use the health information I find on the internet to help me.

□ strongly disagree □ disagree □ neither agree nor disagree　　□ agree □ strongly agree

1. I have the skills I need to evaluate the health resources I find on the internet.

□ strongly disagree □ disagree □ neither agree nor disagree　　□ agree □ strongly agree

1. I can tell of high quality from low-quality health resources on the internet.

□ strongly disagree □ disagree □ neither agree nor disagree　　□ agree □ strongly agree

1. I feel confident using information from the internet to make health decisions.

□ strongly disagree □ disagree □ neither agree nor disagree　　□ agree □ strongly agree

**3、General Health Numeracy Test**

For each question below, follow the directions and read the prompt. Please record your responses on the blank provided for each question.

1）Call your doctor if you have a temperature of 100.4 ºF or greater. The thermometer looks like the following: Do you call the doctor?

Answer： □ Yes □ No

2）If 4 people out of 20 have a chance of getting a cold, what would be the risk of getting a cold?

Answer： _________％

3）Suppose that the maximum heart rate for a 60 year old woman is 160 beats per minute and that she is told to exercise at 80% of her maximum heart rate. What is 80% of that woman’s maximum heart rate?

Answer： ______ beats per minute.

4）You ate half the container of carrots. How many grams of carbohydrates did you eat?

Nutrition Facts

Serving Size: 1 cup (85g) (3 oz.)

Servings Per Container: 2.5

Amount Per Serving

Calories 45 Calories from Fat 0

% Daily Value*

Total Fat 0g 0%

Saturated Fat 0g 0%

Cholesterol 0mg 0%

Sodium 55 mg 2%

Total Carbohydrate 10g 3%

Dietary Fiber 3g 12%

Sugars 5g

Protein 1g

Answer：_________grams

5）Your doctor tells you that you have high cholesterol. He informs you that you have a 10% risk of having a heart attack in the next 5 years. If you start on a cholesterol-lowering drug, you can reduce your risk by 30%. What is your 5-year risk if you take the drug?

Answer： _______％

6）A mammogram is used to screen women for breast cancer. False positives are tests that incorrectly show a positive result. 85% of positive mammograms are actually false positives. If 1,000 women receive mammograms, and 200 are told there is an abnormal finding, how many women are likely to actually have breast cancer?

Answer：____________women

**4、The Cognitive Reflection Test**

1）A bat and a ball cost $1.10 in total. The bat costs a dollar more than the ball. How much does the ball cost?

____ cents

1. If it takes 5 machines 5 min to make 5 widgets, how long would it take 100 machines to make 100 widgets?

____ min

1. In a lake, there is a patch of lily pads. Every day, the patch doubles in size. If it takes 48 days for the patch to cover the entire lake, how long would it take for the patch to cover half of the lake?

____ days

**5、Patient Health Questionnaire (PHQ-9)**

Over the last two weeks, how often have you been bothered by any of the following problems?

1）Little interest or pleasure in doing things.

□ Not at all

□ Several days

□ More than half the days

□ Nearly every day

2）Feeling down, depressed, or hopeless.

□ Not at all

□ Several days

□ More than half the days

□ Nearly every day

3）Trouble falling or staying asleep, or sleeping too much

□ Not at all

□ Several days

□ More than half the days

□ Nearly every day

4）Feeling tired of having little energy.

□ Not at all

□ Several days

□ More than half the days

□ Nearly every day

5）Poor appetite or overeating.

□ Not at all

□ Several days

□ More than half the days

□ Nearly every day

6）Feeling bad about yourself -- or that you are a failure or having let yourself or your family down.

□ Not at all

□ Several days

□ More than half the days

□ Nearly every day

7）Trouble concentrating on things, such as reading the newspaper or watching television.

□ Not at all

□ Several days

□ More than half the days

□ Nearly every day

8）Moving or speaking so slowly that other people could have noticed. Or the opposite -- being so fidgety or restless that you have been moving a lot more than usual.

□ Not at all

□ Several days

□ More than half the days

□ Nearly every day

9）Thoughts that you would be better off dead, or of hurting yourself in some way.

□ Not at all

□ Several days

□ More than half the days

□ Nearly every day

**IV Information and misinformation on the Internet or the mass media**

Based on your current knowledge, quickly determine whether the following statement is correct or not (do not check the Internet for information or ask any person).

1. The ingredients in COVID-19 vaccines are dangerous.

True □ False □

1. The natural immunity I get from being sick with COVID-19 is better than the immunity I get from COVID-19 vaccination.

True □ False □

1. COVID-19 vaccines cause variants.

True □ False □

1. COVID-19 vaccination can give me COVID-19.

True □ False □

1. The mRNA vaccine is not considered a vaccine.

True □ False □

1. COVID-19 vaccines contain microchips.

True □ False □

1. The COVID-19 vaccination will magnetize you.

True □ False □

1. COVID-19 vaccines authorized for use in the United States shed or release their components.

True □ False □

1. The COVID-19 vaccine can change my DNA.

True □ False □

1. A negative COVID test means you are safe.

True □ False □

1. Very high or very low temperature can reduce COVID19 virus.

True □ False □

1. Antibiotics can prevent or treat COVID-19.

True □ False □

1. People who have had COVID-19 and recovered do not need to be vaccinated.

True □ False □
