# Supplementary material for: Factors associated with COVID-19 misinformation rebuttal among college students: a descriptive study
Source: Front Public Health. 2023 Nov 17;11:1233414. doi: 10.3389/fpubh.2023.1233414 (PMC10690778; doi:10.3389/fpubh.2023.1233414)
Supplement: Supplementary file 2 [file Table_1.DOCX]

**Appendix 1.** Means and standard deviations of student responses to the ten myths about COVID19.

| **Myth Statement^a^** | **Valid N.** | **Min. (Correct Response)** | **Max.**  **(Wrong Response)** | **Mean** | **S.D.** |
| --- | --- | --- | --- | --- | --- |
| Myth1  The ingredients in COVID-19 vaccines are dangerous. | 546 | 1 | 2 | 1.22 | 0.42 |
| Myth2  The natural immunity I get from being sick with COVID-19 is better than the immunity I get from COVID-19 vaccination. | 546 | 1 | 2 | 1.27 | 0.44 |
| Myth3  COVID-19 vaccines cause variants. | 546 | 1 | 2 | 1.15 | 0.36 |
| Myth4  The mRNA vaccine is not considered a vaccine. | 546 | 1 | 2 | 1.23 | 0.42 |
| Myth5  COVID-19 vaccines contain microchips. | 546 | 1 | 2 | 1.17 | 0.38 |
| Myth6  COVID-19 vaccines authorized for use in the United States shed or release their components. | 546 | 1 | 2 | 1.18 | 0.39 |
| Myth7  A negative COVID test means you are safe. | 546 | 1 | 2 | 1.54 | 0.50 |
| Myth8  Very high or very low temperature can reduce COVID19 virus. | 546 | 1 | 2 | 1.49 | 0.50 |
| Myth9  Antibiotics can prevent or treat COVID-19. | 546 | 1 | 2 | 1.29 | 0.46 |
| Myth10  People who have had COVID-19 and recovered do not need to be vaccinated. | 546 | 1 | 2 | 1.17 | 0.37 |

^a^ **Sources of myths:** Myths 1-6 were from <https://www.cdc.gov/coronavirus/2019-ncov/vaccines/facts.html> (The Centers for Disease Control and Prevention, USA); Myth 7 was from <https://www.hopkinsmedicine.org/health/conditions-and-diseases/coronavirus/2019-novel-coronavirus-myth-versus-fact> (The Johns Hopkins University School of Medicine); Myths 8 and 9 were from <https://www.who.int/emergencies/diseases/novel-coronavirus-2019/advice-for-public/myth-busters> (the World Health Organisation); Myth 10 was from <https://www.health.gov.au/initiatives-and-programs/covid-19-vaccines/is-it-true> (Australian Department of Health) (last access to all links on 1 August 2022).
