# Supplementary material for: Factors associated with COVID-19 misinformation rebuttal among college students: a descriptive study
Source: Front Public Health. 2023 Nov 17;11:1233414. doi: 10.3389/fpubh.2023.1233414 (PMC10690778; doi:10.3389/fpubh.2023.1233414)
Supplement: Supplementary file 3 [file Table_2.DOCX]

**Appendix 2.** Participant Descriptive Statistics.

| **Predictor Variables^a^** | **Valid N** | **Minimum** | **Maximum** | **Mean** | **S.D.** | **Skewness** | **Kurtosis** |
| --- | --- | --- | --- | --- | --- | --- | --- |
| Years of Higher Education | 546 | 1 | 7 | 2.32 | 0.99 | 0.52 | 0.92 |
| Age | 546 | 17 | 36 | 20.44 | 1.52 | 3.25 | 30.25 |
| Gender (female %) | 546 | 434 (79.5%) | | | | 1.47 | 0.15 |
| Internet Use Frequency | 546 | 1 | 4 | 3.91 | 0.41 | -5.26 | 30.01 |
| Self-Assessed Internet Skill | 546 | 1 | 5 | 3.79 | 1.07 | -0.45 | -0.86 |
| FHL1 | 546 | 1 | 3 | 2.28 | 0.66 | -0.37 | -0.76 |
| FHL2 | 546 | 1 | 3 | 1.79 | 0.68 | 0.30 | -0.87 |
| FHL3 | 546 | 1 | 3 | 2.05 | 0.65 | -0.05 | -0.62 |
| FHL-SUM | 546 | 3 | 10 | 6.14 | 1.37 | 0.22 | 0.59 |
| COHL1 | 546 | 1 | 3 | 1.53 | 0.66 | 0.84 | -0.40 |
| COHL2 | 546 | 1 | 3 | 1.74 | 0.67 | 0.36 | -0.80 |
| COHL3 | 546 | 1 | 3 | 1.83 | 0.67 | 0.22 | -0.81 |
| COHL-SUM | 546 | 3 | 9 | 5.10 | 1.65 | 0.68 | 0.11 |
| CRHL1 | 546 | 1 | 3 | 2.02 | 0.68 | -0.03 | -0.85 |
| CRHL2 | 546 | 1 | 3 | 1.87 | 0.66 | 0.14 | -0.71 |
| CRHL3 | 546 | 1 | 3 | 1.82 | 0.68 | 0.23 | -0.84 |
| CRHL4 | 546 | 1 | 3 | 2.03 | 0.60 | -0.01 | -0.25 |
| CRHL5 | 546 | 1 | 3 | 1.93 | 0.61 | 0.03 | -0.30 |
| CRHL6 | 546 | 1 | 2 | 1.45 | 0.50 | 0.20 | -1.97 |
| CRHL-SUM | 546 | 6 | 17 | 11.13 | 2.65 | 0.04 | 0.06 |
| eHEALS1 | 546 | 1 | 5 | 3.56 | 0.92 | -1.05 | 1.35 |
| eHEALS2 | 546 | 1 | 5 | 3.48 | 0.92 | -0.92 | 1.00 |
| eHEALS3 | 546 | 1 | 5 | 3.50 | 0.90 | -0.95 | 1.14 |
| eHEALS4 | 546 | 1 | 5 | 3.53 | 0.89 | -1.00 | 1.24 |
| eHEALS5 | 546 | 1 | 5 | 3.59 | 0.89 | -1.20 | 1.67 |
| eHEALS6 | 546 | 1 | 5 | 3.53 | 0.87 | -1.00 | 1.23 |
| eHEALS7 | 546 | 1 | 5 | 3.61 | 0.86 | -1.14 | 1.83 |
| eHEALS8 | 546 | 1 | 5 | 3.48 | 0.89 | -0.88 | 0.86 |
| eHEALS-SUM | 546 | 8 | 40 | 28.29 | 6.04 | -1.07 | 2.28 |
| GHNT1 | 546 | 1 | 2 | 1.31 | 0.46 | 0.84 | -1.31 |
| GHNT2 | 546 | 1 | 2 | 1.36 | 0.48 | 0.60 | -1.65 |
| GHNT3 | 546 | 1 | 2 | 1.41 | 0.49 | 0.35 | -1.88 |
| GHNT4 | 546 | 1 | 2 | 1.77 | 0.42 | -1.28 | -0.36 |
| GHNT5 | 546 | 1 | 2 | 1.51 | 0.50 | -0.05 | -2.01 |
| GHNT6 | 546 | 1 | 2 | 1.54 | 0.50 | -0.16 | -1.98 |
| GHNT-SUM | 546 | 6 | 12 | 8.90 | 1.88 | 0.10 | -1.17 |
| CRT1 | 546 | 1 | 2 | 1.75 | 0.43 | -1.14 | -0.70 |
| CRT2 | 546 | 1 | 2 | 1.55 | 0.50 | -0.18 | -1.97 |
| CRT3 | 546 | 1 | 2 | 1.59 | 0.49 | -0.37 | -1.87 |
| CRT-SUM | 546 | 3 | 6 | 4.88 | 1.08 | -0.43 | -1.16 |
| PHQ-SUM | 546 | 0 | 27 | 5.86 | 5.56 | 1.13 | 1.52 |

^a^ Years of HE: Years of Higher Education; FHL-SUM: functional health literacy sum; COHL-SUM: communicative health literacy sum; CRHL-SUM: critical health literacy sum; eHL-SUM: digital health literacy sum; GHNT-SUM: General Health Literacy Test sum; CRT-SUM: Cognitive Recognition Test sum; PHQ-SUM: PHQ-9 Patient Depression Questionnaire sum.
